# Oral Mucosa Status and Saliva Parameters of Multimorbid Adult Patients Diagnosed with End-Stage Chronic Kidney Disease

**DOI:** 10.3390/ijerph182312515

**Published:** 2021-11-27

**Authors:** Agata Trzcionka, Henryk Twardawa, Katarzyna Mocny-Pachońska, Rafał Korkosz, Marta Tanasiewicz

**Affiliations:** Department of Conservative Dentistry with Endodontics, Faculty of Medical Sciences in Zabrze, Medical University of Silesia, Plac Akademicki 17, 41-902 Bytom, Poland; htwardawa@sum.edu.pl (H.T.); kpachonska@sum.edu.pl (K.M.-P.); rkorkosz@sum.edu.pl (R.K.); martatanasiewicz@sum.edu.pl (M.T.)

**Keywords:** chronic diseases, end-stage chronic kidney disease, diabetes mellitus, hypertension, saliva, mucosa

## Abstract

There has been an increasing number of patients diagnosed with chronic diseases. Many of those diseases determine changes in patients’ social and even professional life. The aim of the present work was to analyze saliva and oral mucosa parameters in adult patients diagnosed with chronic diseases. A total of 228 patients took part in the research. A total of 180 patients were hemodialyzed in Diaverum dialysis stations, and there were 48 patients from the Conservative Dentistry with Endodontics Clinic of the Academic Centre of Dentistry of Silesian Medical University in Bytom and patients from the dentistry division of the Arnika Clinic in Zabrze not diagnosed with any such diseases. Selected saliva parameters (secretion, pH and buffer capacity) and mucosa status were examined. In order to obtain information regarding xerostomia, patients were given a questionnaire. Patients assigned to the control group rarely used water to make swallowing easier or used candies to lessen the feeling of oral cavity dryness. They also rarely suffered from eye dryness. The feeling of dryness also did not influence their social life. The amount of produced saliva was higher in the control group. There was a significantly lower percentage of patients with a low buffer capacity of saliva. There were no differences in terms of saliva pH values between the two groups of patients. A significantly lower number of patients were diagnosed with ecchymosis, candidiasis, scrotal tongue and mechanical damages. Patients diagnosed with chronic diseases need to be provided with long-term care.

## 1. Introduction

An increasing number of people are being diagnosed with chronic diseases. Many of those diseases determine changes in patients social and even professional life. Very often, chronic diseases are incurable, long-term conditions, and their treatment is mostly concentrated on dealing with their symptoms [1]. The unpredictability of the illness course undoubtedly influences patients’ quality of life. Patients very often describe fear and worries when describing their life [1]. 

Multimorbidity affects patients’ social relations and mental health [2]. It is still a challenge to implement a healthy lifestyle and a strategy that would be helpful in reducing the effects of chronic diseases [3]. It needs to be remembered that oral diseases may influence the course and treatment of the general disease, and they undoubtedly influence patients’ quality of life. Proper oral status would make an impact on social relations, proper food intake and self-esteem, and all of these factors are strongly connected to a satisfactory professional life. 

An increasing number of elderly people are being diagnosed with chronic diseases, and very often, they also suffer from oral pathologies. The burden of oral diseases has a negative effect on older peoples’ quality of life [4].

The following oral findings have been described as characteristic of people with end-stage chronic kidney disease: xerostomia [5], uremic stomatitis, ulcerations, geographic tongue, *petechiae*, *ecchymoses* and bleeding [5,6,7,8,9,10,11,12]. In that group of patients, there is also decreased salivary secretion, and an increase in its density is observed [9,13,14]. Very often, patients also complain about taste disorders [15,16] and halitosis [17,18,19,20].

In the oral cavity of patients with hypertension, pathologies strongly related to the taken medicaments can be observed. Most frequently, drug-induced gingival overgrowth can be observed [21,22,23,24,25,26]. Changes in the oral cavity can be observed after 1–3 months from the beginning of therapy. Very often, patients also suffer from dry mouth syndrome and burning mouth syndrome [27,28].

In patients diagnosed with diabetes, it is possible to observe changes in the salivary gland structure [29,30] and xerostomia [31]. The saliva of diabetics is characterized by a higher concentration of glucose, decreased pH, lower buffer capacity and higher viscosity [32,33,34,35]. Diabetes increases the risk of filiform and fungiform papillae atrophy, which may lead to pain and burning [34,35]. A higher risk of candidiasis is also observed (most often, the isolated species are *Candida albicans, Candida glabrata, Candida tropicalis* and *Candida parapsilosis*) [36,37,38,39]. Diabetes is also mentioned as a reason for lichen planus occurrence [38].

Due to the fact that chronic kidney disease, hypertension and diabetes very often coexist, we decided to analyze saliva and oral mucosa parameters in adult patients diagnosed with the mentioned chronic diseases. 

## 2. Materials and Methods

The authors were invited to take part in epidemiological research aimed at assessing the oral cavity status of hemodialyzed patients. The research was organized by Diaverum hemodialysis stations. Our contribution to the project was the examination of patients treated in Katowice, Kraków, Głubczyce and Warszawa. A total of 228 patients were included in the study. A total of 180 patients were hemodialyzed in Diaverum dialysis stations, and 48 were treated in the Conservative Dentistry with Endodontics Clinic of the Academic Centre of Dentistry of the Medical University of Silesia in Bytom and in the Dentistry Division of the Arnika Clinic in Zabrze [40,41].

### 2.1. Inclusion Criteria

#### 2.1.1. Examined Group

The examined group consisted of patients over 40 years old with diagnosed end-stage chronic kidney disease, blood hypertension and/or diabetes for at least 2 years and who had given written approval to take part in the research.

#### 2.1.2. Control Group

The control group consisted of patients over 40 years old who were not diagnosed with any civilization disease from the examined group and who had given written approval to take part in the research.

### 2.2. Exclusion Criteria

Patients were excluded if they did not agree to take part in the research, did not demonstrate a will to cooperate, were pregnant, were in a state of exacerbated general disease, were incapacitated or had suffered from civilization diseases for less than 2 years.

The examined group (EG) consisted of 180 patients treated in Diaverum dialysis stations in Katowice, Głubczyce, Warszawa and Kraków, who were diagnosed with end-stage chronic kidney disease. Patients were considered to have blood hypertension based on at least one of the blood tension values being equal to or higher than 140/90 mmHg in a few measurements over the course of at least two medical check-ups. Patients with diabetes were diagnosed on the basis of World Health Organization (WHO) recommendations with a fasting plasma glucose test and impaired glucose tolerance (IGT) test. Patients were divided into four subgroups on the basis of the general disease that they suffer from:

R—patients diagnosed with chronic kidney disease (42 people).
+ H—patients diagnosed with chronic kidney disease and hypertension (79 people).
+ D—patients diagnosed with chronic kidney disease and diabetes (16 people).
+ H + D—patients diagnosed with chronic kidney disease, hypertension and diabetes (43 people).

The control group (CG) was composed of 48 patients from the Conservative Dentistry with Endodontics Clinic of the Academic Centre of Dentistry of the Medical University of Silesia in Bytom and patients that, to date, were not diagnosed with any of the civilization diseases from the examined group. 

The examination of hemodialyzed patients took place at the hemodialysis stations. Patients were informed how to prepare for the examination and asked to arrive earlier. In the first stage, the oral cavity assessment was carried out. Hemodialysis lasted 4–5 h, and during that time, the questionnaire examination was conducted. In that part, the following questions regarding xerostomia and the feeling of thirst were asked:

- I drink water while swallowing the food (never, sometimes, often).
- I feel dryness in oral cavity while eating meals (never, sometimes, often).
- Feeling of dryness wakes me up and I drink at night (never, sometimes, often).
- I feel discomfort while eating dry meals (never, sometimes, often).
- I suck candies to decrease the feeling of dryness (never, sometimes, often).
- I have difficulties in swallowing food (never, sometimes, often).
- My skin on the face is dry (never, sometimes, often).
- My eyes are dry (never, sometimes, often).
- My lips are dry (never, sometimes, often).
- I often fell thirst, what is problematic (never, sometimes, often).
- I feel thirst during the day (never, sometimes, often).
- I feel thirst during the night (never, sometimes, often).
- I take thirst into consideration when planning my free time (never, sometimes, often).
- I feel thirst before hemodialysis (never, sometimes, often).
- I feel thirst during hemodialysis (never, sometimes, often).
- I feel thirst after hemodialysis (never, sometimes, often).

Only hemodialyzed patients were asked the last three questions.

In the case of the CG, the examination was carried out during patient check-ups. Before the appointment, patients received information on how to prepare. After the oral cavity examination, patients received questionnaires and were asked to fill them in (apart from the last three questions).

Saliva examination was carried out with the usage of *Saliva-Check Buffer Kit (GC)*. Patients were asked not to drink, eat, chew gum or brush their teeth for one hour before the examination.

GC is composed of in vitro pH test strips, saliva dispensing cups, wax gum pieces for saliva stimulation, saliva dispensing pipettes and buffer test strips.

The examination was composed of the following:

- Stimulated saliva secretion—the aim of the examination was to calculate the salivary flow rate (SFR). Patients were asked to chew a paraffin cube (1g) for 60 s and to swallow the saliva. In the next stage of the examination, while chewing the same paraffin cube, participants deposited saliva into the sterile test tube for 5 min. Salivary flow rate was calculated in ml/min. Patients who underwent hemodialysis were examined before and after the procedure.
- Buffer capacity of saliva—with the usage of a pipette, one drop of gathered saliva was placed onto each of the three test pads. After 2 min, the color of each pad was compared with the table provided by the producer. Each color was given a particular number of points that were added together, and on the basis of the recorded results, buffer capacity was classified as very low (0–5), low (6–9) or normal (10–12).
- Saliva pH—the pH strip was placed in the sample of saliva obtained from the patient for 10 s. The obtained result was compared with the dental saliva pH indicator provided by the producer.

Oral mucosa was examined in order to note the occurrence of the following pathologies: ulcerations, white patches, red patches, malformations, ecchymosis, candidiasis, herpes simplex, geographic tongue, fissured tongue, the smell of acetone, signs of previous operations and trauma-related lesions. If any of the pathologies were observed, their location was also noted. Patients were also asked if they feel any pain, burning mouth syndrome, dry mouth syndrome or taste disorders. The overgrowth of gingiva with regard to the medications taken by the participant was also looked for.

### 2.3. Statistical Analysis

The *Kruskal–Wallis* test was used to assess differences in the averages of the parameters between the groups. In the case of significant differences between the averages, the *Mann–Whitney U* test was performed to verify the accuracy of these differences. The chi-squared test was used to analyze the qualitative variables. If statistically significant differences were observed, the Eta effect was calculated. 

All of these tests were performed using IBM’s SPSS Statistics 23 program (IBM, Armonk, NY, USA), and *p* < 0.05 was considered indicative of a statistically significant result.

## 3. Results

The control group was composed of 15 males and 33 females, and their average age was 52.71. A total of 17 females and 25 males were diagnosed only with end-stage chronic kidney disease (the average age was 67.21). In the R + H subgroup, there were 48 male and 31 female patients, and their average age was 62.54. The average age in the R + D group was 70.16 (10 males and 6 females). The R + H + D group was composed of 27 males and 16 females, whose average age was 72.86 [41].

The frequency and percentage distributions of the answers to the questions regarding xerostomia are presented in Table 1. 

Patients from the control group least often declared drinking water to make the swallowing of meals easier, while in hemodialyzed patients, those additionally diagnosed with both diabetes and hypertension declared it the most often. Regarding the most frequent answer to the question about the feeling of dryness in the mouth while eating, the most frequent answer in the control group was ‘never’ and only in the R + D group was this the same, a high number of patients answered ‘never’ and ‘sometimes’. In each of the examined groups, most of the patients did not feel discomfort while eating dry products. Patients from the control group least often declared eating hard candies. Most of the patients from each subgroup of the examined groups never had a problem when swallowing food. Patients from the control group least often complained about having dry eyes in comparison to hemodialyzed patients, among whom dry eyes were most often declared by patients from the R + H group and the least often by patients from the R group. Most of the patients from each group declared that they never had the feeling of dry lips.

### 3.1. Feeling of Thirst

Regarding the analysis of the obtained answers with the *chi-square* test, statistically significant differences were observed in four out of seven questions. The frequency and the percentage distributions of the answers are presented in Table 2. The feeling of thirst was not problematic for patients from the control group, and in the examined group, it was most often declared by patients from the R + H and R + H + D subgroups. Healthy patients least often felt thirsty both at night and during the day. In the examined group, that complaint was observed in patients with hypertension and those with both hypertension and diabetes. Patients from the control group least often declared that the feeling of thirst influenced their social life. In the hemodialyzed patients, those with hypertension and with both hypertension and diabetes most often complained that the feeling of thirst influenced their social life.

### 3.2. Saliva Parameters

#### Stimulated Saliva 

**Figure 1:**
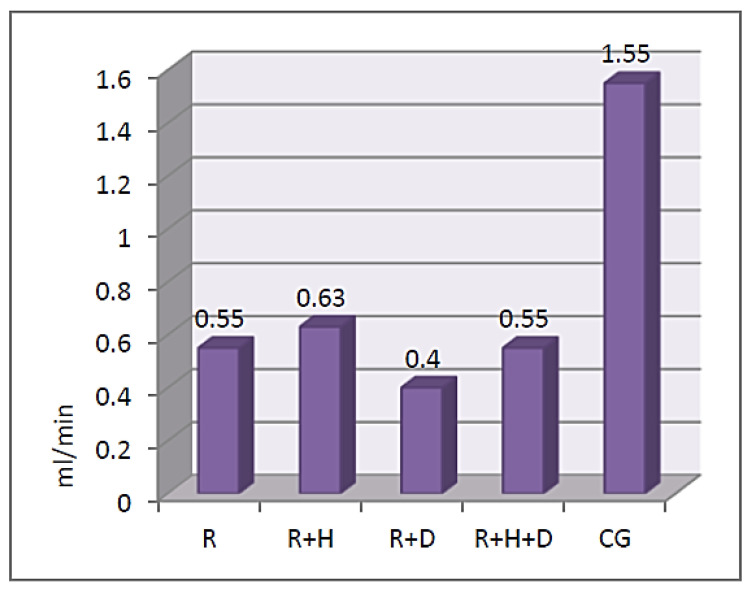
The average values of stimulated saliva secretion before hemodialysis.

In patients from the examined group, stimulated saliva was assessed twice, before and after hemodialysis, while in the control group, it was only assessed once. The results of stimulated saliva secretion in the control group ranged from 0.05 to 4.5 mL/min. In the examined group, those values assessed before hemodialysis were R—0.05–2.25 mL/min, R + H—0–2.75 mL/min, R + D—0.02–1 mL/min and R + H + D—0.05–1.5 mL/min, while after hemodialysis, those values were R—0–5.5ml/min, R + H—0–2.5ml/min, R + D—0.3–2.4ml/min and R + H + D—0–2ml/min. Figure 1 presents the average values of stimulated saliva secretion before hemodialysis (*M* = 1.55; *SD* = 0.86 vs. *M* = 0.55; *SD* = 0.38 and *M* = 0.63; *SD* = 0.55 and *M* = 0.40; *SD* = 0.31 and *M* = 0.55; *SD* = 0.55) (ε^2^ = 0.27).

**Figure 2:**
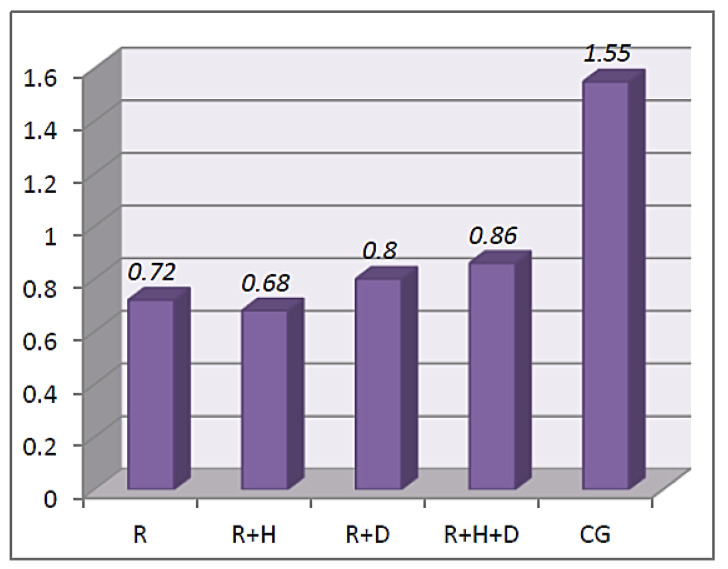
The average amount of stimulated saliva measured after hemodialysis.

The analysis of the results of the *Kruskal–Wallis* test proved that there were statistically significant differences between the groups of patients in the amount of stimulated saliva before hemodialysis χ^2^(4) = 60.42; *p* < 0.001; ε^2^ = 0.27. The *U Mann–Whitney* test proved that the average amount of stimulated saliva was observed in the control group in comparison to the examined one (before and after hemodialysis). There were statistically significant differences between the groups with regard to the amount of stimulated saliva measured after hemodialysis χ^2^(4) = 39.71; *p* < 0.001; ε^2^ = 0.29. The *U Mann–Whitney* tests proved that the average amount of stimulated saliva in patients from the control group was significantly higher than in patients from the examined one (R, R + H, R + D and R + H + D) (*M* = 1.55; *SD* = 0.86 vs. *M* = 0.72; *SD* = 0.55 and *M* = 0.68; *SD* = 0.59 and *M* = 0.80; *SD* = 0.64 and *M* = 0.86; *SD* = 1.00) (ε^2^ = 0.27) (Figure 2). 

### 3.3. Buffer Capacity

**Figure 3:**
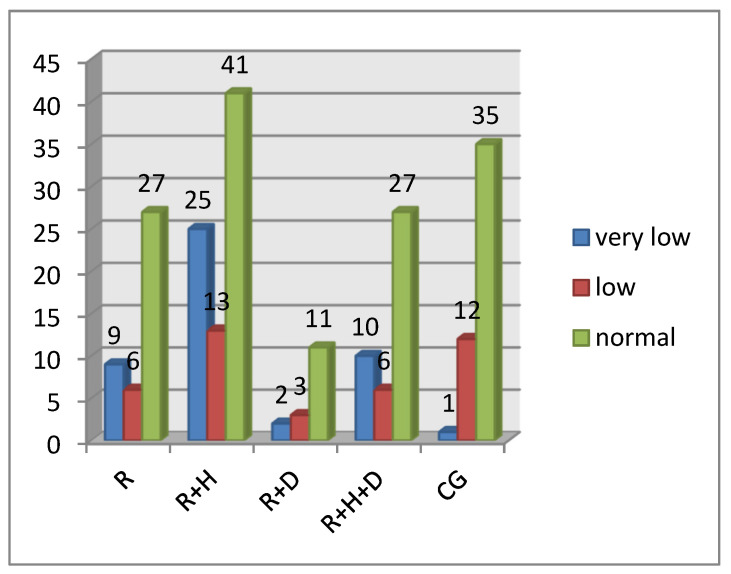
The distribution of saliva buffer capacity in particular groups.

In patients from the control group, a very low buffer capacity was observed in 2.1% of patients, low in 25% and normal in 72.9%. In the R group, a very low buffer capacity was diagnosed in 21.4% of patients, low in 14.3% and normal in 64.3%. Hemodialyzed patients with hypertension (R + H) had very low buffer capacity in 31.6% of patients, low in 16.5% and normal in 51.9%. In the R + D group, a very low buffer capacity was observed in 12.5% of patients, low in 18.8% and normal in 68.8%. In the R + H + D group, a very low buffer capacity was observed in 23.3% of patients, low in 14% and normal in 62.8%. Figure 3 presents the distribution of saliva buffer capacity in the particular groups. 

The results of the *chi-squared* test proved that there were statistically significant differences in the buffer capacity of saliva between particular groups of patients χ^2^(8) = 17.87; *p* < 0.05; η = 0.23. In the control group, there was a lower percentage of people with very low buffer capacity in comparison with hemodialyzed patients. 

### 3.4. Saliva pH

In the control group, the minimal value of pH was 6.4 and the maximum 7.8. In each subgroup of the examined group, the obtained pH values ranged from 5 to 7.8. The obtained results were analyzed with the *Kruskal–Wallis* test. The results are presented in Table 3.

**Figure 4:**
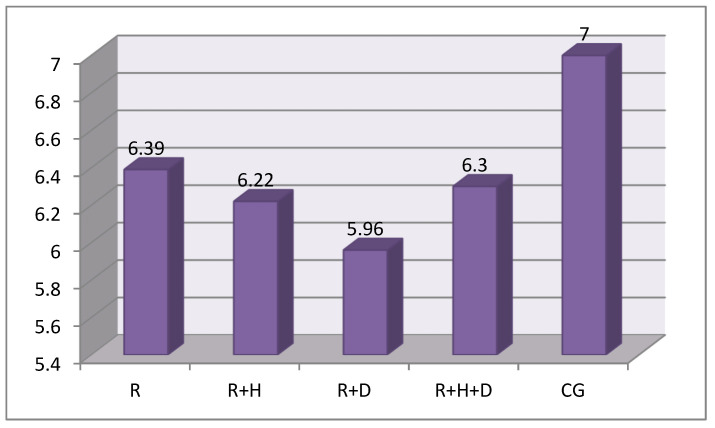
The average values of saliva pH in groups.

The concentration of hydrogen ions was calculated with the following formula: pH = −log[H^+^]. The concentration of hydrogen ions was similar in all groups. The following average values of pH were noted: R—6.39; R + H—6.22; R + D—5.96; R + H + D—6.3; and CG—7 (Figure 4).

Despite the fact that there were no statistically significant differences, the highest values of pH were noted in the control group. In the examined one, the saliva pH ranged from 5.96 in the R + D group to 6.39 in the R group.

### 3.5. Mucosa Status

The results of the *chi-squared* test for pathologies observed in mucosa are presented in Table 4. 

**Figure 5:**
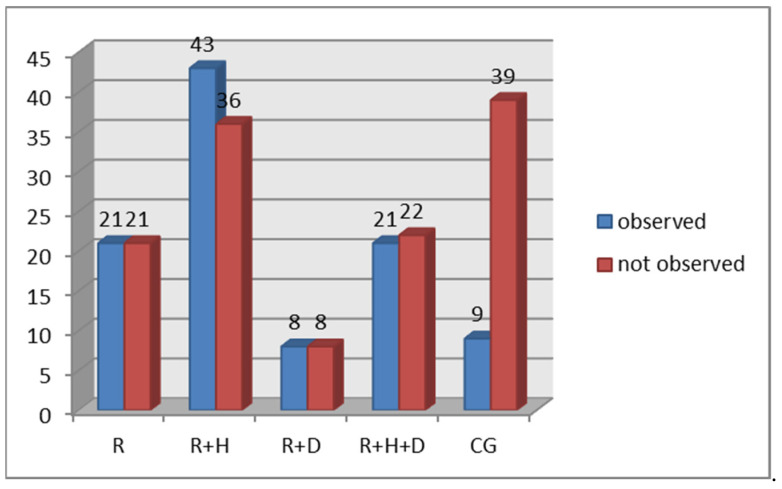
Occurrence of dryness in particular groups.

The percentage of patients who declared that they felt dryness in the oral cavity was significantly lower in the control group than in the hemodialyzed groups (Figure 5).

**Figure 6:**
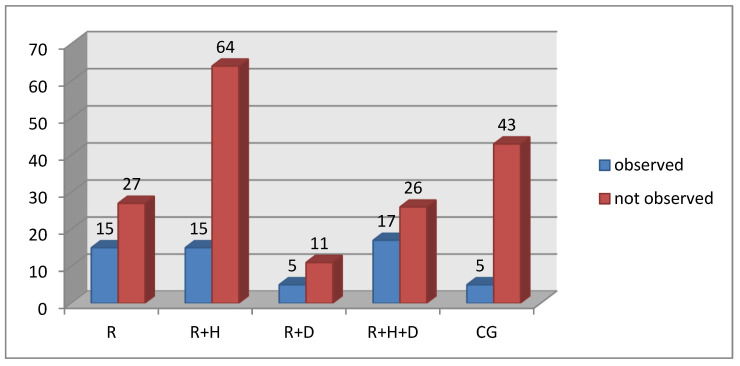
Occurrence of ecchymosis in particular groups.

The percentage of patients with diagnosed ecchymosis was significantly lower in the control group than in all subgroups of the examined group. In hemodialyzed patients, the R + H group had the lowest percentage of patients with ecchymosis (Figure 6).

**Figure 7:**
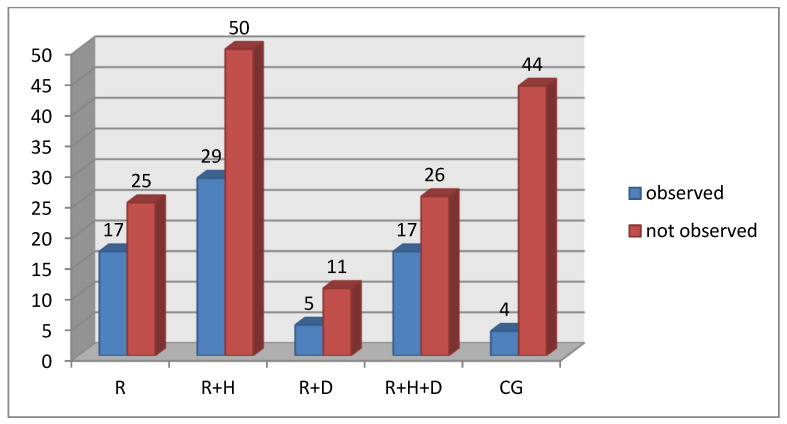
Occurrence of candidiasis in particular groups.

Similarly, the percentage of patients with *candidiasis* was significantly lower in the control group in comparison to the examined one (Figure 7). 

**Figure 8:**
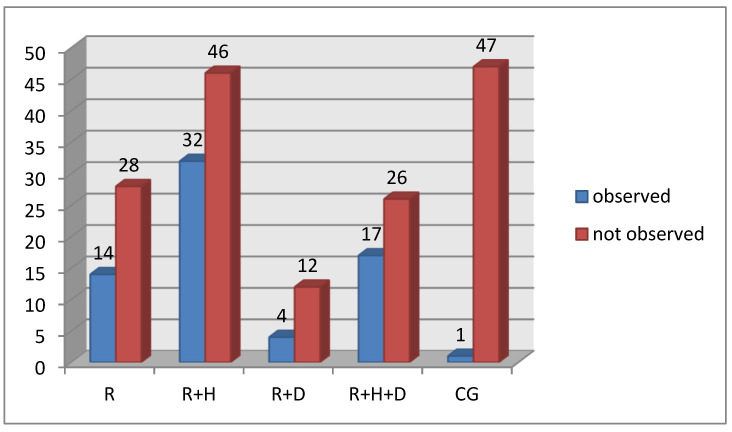
Occurrence of fissured tongue in particular groups.

While assessing the frequency of occurrence of fissured tongue, it was observed that the percentage of patients with that pathology was significantly lower in the healthy patient group than in the hemodialyzed patient groups (Figure 8).

**Figure 9:**
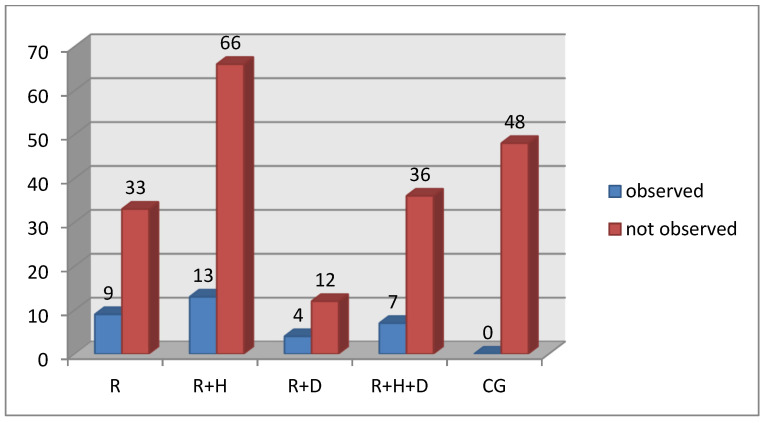
Occurrence of trauma-related oral lesions in particular groups.

Statistically significant differences were observed in the assessment of trauma-related oral lesions. The percentage of patients with that type of lesion was significantly lower in the control group than in the examined one (Figure 9).

The analysis of the occurrence of other pathologies in patients diagnosed with chronic kidney disease showed no cases of ulceration, *herpes simplex* or overgrowth of gingiva, and none had signs of operations in the oral cavity. In 15 patients (36%), pedunculated malformations of the cheek’s mucosa were observed. White patches of the oral mucosa were observed in seven patients (17%), and they were localized in the retromolar triangle region. A total of four patients (10%) declared taste disorders. Geographic tongue was observed in three patients (7%), and the same number of patients declared halitosis. In two patients (5%), red patches of the oral mucosa were observed—on the soft palate and on the oral mucosa of the right cheek. One patients felt pain and one felt burning mouth syndrome. 

In patients from the R + H group, the least often observed pathologies were overgrowth of gingiva, signs of operations, herpes simplex, ulceration and burning mouth syndrome—each pathology was observed in one patient (five different people). In 15 patients (19%), malformations of the cheek’s oral mucosa and white patches (cheek and soft palate) were observed. Eight people (10%) declared taste disorders. In six patients (8%), red patches (cheek) were observed. Geographic tongue and halitosis were diagnosed with the same frequency—in four patients (5%). Three patients (4%) felt pain.

In patients diagnosed with chronic kidney disease and diabetes, there were no cases of pain or mouth burning syndrome, and no signs of previous operations were observed. In one patient, red patches (oral mucosa of the right cheek), geographic tongue and overgrowth of oral mucosa were observed. Two patients were diagnosed with halitosis and white patches on the oral mucosa of the cheek. Three patients (19%) observed taste disorders.

None of the following pathologies were observed in patients from the R + H + D group: overgrowth of gingiva, signs of previous operations, herpes simplex or ulcerations. One patient (2%) was diagnosed with halitosis, and one felt pain. Two patients (5%) suffered from burning mouth syndrome. Geographic tongue was diagnosed in Three patients (7%). Five patients had red patches on the cheek’s mucosa. Eight people (19%) declared taste disorders. In 10 patients (23%), white patches on the cheek’s mucosa were observed. Malformations of mucosa were observed in 17 patients (40%).

None of the patients from the control group were diagnosed with pain, mouth burning syndrome, halitosis or overgrowth of gingiva, and none had any signs of previous operations. One patient had geographic tongue. In two patients (4%), ulcerations were observed. Herpes simplex was observed in three patients (6%). The same number of patients declared taste disorders. In four patients (8%), red and white patches localized on the cheek’s mucosa were observed, and in five patients (10%), malformations of the oral mucosa were observed.

## 4. Discussion

Multimorbid adult patients have to struggle with many oral conditions that very often influence many aspects of their lives. It is crucial to know the exact problem of the patients diagnosed with common chronic diseases to be able to improve their quality of life [1,2,3].

### 4.1. Secretion of Saliva

The proper amount and the composition of saliva are crucial to maintain the optimal health of the oral cavity. The general diseases discussed in this research influence the probability of saliva disorder occurrence. The decreased secretion of saliva (*sialopenia, hyposialia*) leading to the drying out of the oral mucosa is called *xerostomia*. With regard to the causes of the dryness, for true xerostomia *(vera, primaria*)—caused by salivary gland disorders that are the result of local or systemic disease and symptomatic xerostomia (*spuria, symptomatica*)—no changes in salivary glands are observed, and for the cause, emotional states or taken medications can be disregarded. The specific causes of xerostomia in hemodialyzed patients are the disturbance of water–electrolyte homeostasis; the restricted intake of fluids, which additionally increases saliva’s viscosity, sodium, phosphate and urea concentrations; and increased pH. Very often, all those disorders make it impossible to eat, speak (without drinking water) or use removable dentures and may lead to uremic breath and taste disorders [13,17,19].

In our own research, we observed that stimulated saliva secretion was significantly higher in healthy patients in comparison to the hemodialyzed patients. A lower secretion of saliva in hemodialyzed patients was observed by other researchers [6,8,16,18,42,43]. We did not observe statistically significant differences in the amount of saliva with regard to diabetes; however, it should be noted that the values obtained in the R + D group were the lowest (the average value of simulated saliva secretion was 0.4 mL/min). Kaushik et al. compared the stimulated and unstimulated saliva secretion in healthy and hemodialyzed patients. They obtained statistically lower results in patients diagnosed with end-stage chronic kidney disease for both types of saliva (in the examined group: unstimulated saliva—0.31+/−0.01 mL/min and stimulated—0.66+/−0.02 mL/min; in the control group: unstimulated saliva—0.52+/−0.06 mL/min and stimulated—1.16+/−0.11 mL/min) [44]. Similar results were presented by Teratani et al., who observed lower average values in hemodialyzed patients. Even though they did not observe statistically significant differences, they found that in hemodialyzed patients with diabetes, the amount of saliva was lowest (0.7 mL/min) in comparison to hemodialyzed patients without diabetes (0.9 mL/min) [42]. Significantly lower average values of stimulated saliva in hemodialyzed patients with diabetes (1.2 mL/min) in comparison to hemodialyzed patients without diabetes (1.6 mL/min) were also observed by Vesterinen et al. [43]. The discussed results of our own and other authors’ research suggest that the coexistence of diabetes decreases the amount of saliva secretion. No information regarding the influence of hemodialysis on saliva secretion was found in the literature. Our own results showed that the amount of saliva increases after hemodialysis; however, it was still lower than in healthy patients. In the literature, we can find the results of an analysis of the influence of hemodialysis on saliva pH. *Wilczyńska-Borawska* conducted her research on 80 patients and observed a decrease in saliva pH (unstimulated saliva) in the last hour of hemodialysis, from 8.0 to 7.15 [45]. Swapna et al. compared unstimulated saliva pH with regard to diabetes coexistence in hemodialyzed patients. They observed that the average pH values in patients without diabetes were 7.14+/−1.18 and were significantly higher when compared to patients with diabetes—7.02+/−1.19 [16]. Kaushik et al. assessed stimulated and unstimulated saliva pH. They obtained significantly higher values of unstimulated saliva pH in hemodialyzed patients (7.24+/−0.25) in comparison to the control group (6.60+/−0.32); a pH assessment of the stimulated saliva did not show any statistically significant differences (examined group: 7.28+/−0.25; control group: 7.24+/−0.25). They explained that it was the increase in sodium and hydrocarbonate secretion with the increase in saliva secretion that led to an increase in pH [44]. In our research, we assessed the stimulated saliva pH before hemodialysis. No statistically significant differences between the examined and control groups were observed. However, the lowest values were observed in hemodialyzed patients with diabetes. Our results are contradictory to those obtained by *Swapana* and *Borawska-Wilczyńska* [16,45]. The pH values observed by *Swapana* were higher than 7 and those by *Borawska-Wilczyńska* were even higher at 8, while the values observed by us were lower than 6.5. This may be a result of patients not following the instructed restrictions before the examination; we were not able to check if the patients had empty stomachs. 

### 4.2. Buffer Capacity of Saliva

The buffer capacity of saliva was also examined. We observed a significantly lower percentage of patients with low buffer capacity in the control group in comparison to the hemodialyzed ones. Most of the patients from both groups had a high buffer capacity of saliva. Kaushik et al. assessed the buffer capacity of stimulated saliva in hemodialyzed and healthy patients and found no statistically significant differences between those patients. They obtained statistically significant differences between those values for unstimulated saliva; the buffer capacity in hemodialyzed patients was higher. They explained the lack of differences for stimulated saliva as being a result of the increase in carbohydrate concentrations in the parotid gland caused by stimulation itself, which must have led to an increase in buffer capacity. Those arguments make it necessary to consider if the methodology of the buffer capacity examination was appropriate, as the described process could influence our results [44].

### 4.3. Dry Mouth Syndrome

Patients diagnosed with end-stage chronic kidney disease very often suffer from dry mouth syndrome. Our own results of stimulated saliva secretion showed a significantly lower amount of saliva in the examined group. The obtained results proved that the saliva secretion in our patients was very low, which may lead to dry mouth syndrome. To examine the problem of xerostomia, patients were asked to answer a questionnaire. The results of that part of our examination proved that hemodialyzed patients most often have to drink water while eating, use candies to make the feeling of dryness less irritating and complain about eye’s dryness. Patients from the examined group also more frequently complained about feeling thirst—it was problematic for them, they felt it at night and during the day, and it influenced their social life. We also observed that the percentage of patients who complained about xerostomia was significantly lower in the control group when compared to the hemodialyzed ones. Similar results were obtained by Ahmed et al. [10], Patil et al. [11], Yadav et al. [12] and Oyetola et al. [9]. Teratani et al. asked similar questions and observed that hemodialyzed patients (with and without diabetes) complained more often than healthy patients about dry mouth, problems when eating dry food, eye dryness and stickiness and a hoarse voice [42]. It can be concluded that the coexistence of end-stage chronic kidney disease influences the frequency of dry mouth syndrome occurrence, which is confirmed by the results of other authors.

### 4.4. Pathologies in Oral Mucosa

The analysis of pathologies in oral mucosa showed statistically significant differences in the occurrence of ecchymosis, fissured tongue, *candidiasis* and trauma-related oral lesions. The frequency of the occurrence of these pathologies was significantly lower in healthy patients in comparison to hemodialyzed patients. The increased frequency of *candidiasis* in hemodialyzed patients was observed by Oyetola et al., which they explained as being a result of malnutrition, a restrictive diet and the intake of immunosuppressants [9]. Ahmed et al. observed that same pathology in both healthy and hemodialyzed patients [10]. Oyetola et al. mentioned that *candidiasis* may be more frequent in hemodialyzed patients due to xerostomia, decreased saliva secretion, not maintaining proper oral hygiene, the coexistence of diabetes and the intake of medications that may influence saliva secretion [9]. Yadav et al. observed the discussed pathology in four hemodialyzed patients (8%) and no healthy patients [12]. The obtained results confirmed those discussed above.

Several teams of researchers examined oral mucosa and the occurrence of ecchymosis [10,11,12,16]. Ahmed et al. observed this pathology in 20% of hemodialyzed patients [10], while Yadav et al. only in one patient with end-stage chronic kidney disease and in no healthy patients [12]. These results confirmed our observations. A higher susceptibility to ecchymosis in hemodialyzed patients is explained by the general disease, which causes changes in platelet aggregation, and the influence of hemodialysis, which decreases the number of thrombocytes due to their mechanic damage and heparin intake [10].

Yadava et al. assessed oral mucosa with regard to fissured tongue occurrence [12]. They did not observe this pathology in the examined group, which is the opposite to our results. Our own results with regard to trauma-related pathologies may be explained by the lower amount of saliva in hemodialyzed patients, which increases the risk of mechanical injuries.

### 4.5. Halitosis

In our own research, no statistically significant differences were observed with regard to halitosis between patients. However, it has to be noted that none of the patients from the control group declared that problem, while in particular subgroups of the examined group, the following number of patients had that problem: R—three (7%), R + H—four (5%), R + D—two (12%) and R + H + D—one (2%). Oyetola et al. observed a statistically higher number of halitosis in hemodialyzed patients (12%) when compared to healthy people [9]. Similar results were obtained by Yadav et al.—31% of hemodialyzed patients suffered from fetor ex ore [12]. Ahmed et al. observed that same pathology in 66% of hemodialyzed patients and only in 2% of healthy people. They also found a positive correlation of halitosis with xerostomia [10]. Similar results were presented by Patil et al.; in hemodialyzed patients, 34% were diagnosed with halitosis, while in healthy ones, 14% were diagnosed [11]. Souza et al. concluded that halitosis was less often observed in patients after transplantation in comparison to those who were still waiting for the transplantation to be performed [46]. The problem of halitosis in hemodialyzed patients with and without diabetes was examined by Swapna et al., who observed a higher frequency of this pathology in patients without diabetes [16]. Our own results suggest that end-stage chronic kidney disease makes the possibility of halitosis occurrence higher.

### 4.6. Taste Disorders

The presence of urea and its decomposition to ammonia and carbon dioxide in the saliva of hemodialyzed patients are regarded as being responsible for the metallic taste observed in that group of patients. Taste disorders may also be caused by metabolic disorders, taken medicaments, changes in saliva composition and a decrease in its secretion and a low level of zinc [8,9,10,13]. In our own research, no statistically significant differences in taste disorders were observed. However, it needs to mentioned that it was more often observed in hemodialyzed patients, which was also observed by other authors [10,11,12].

### 4.7. Limitations of the Work

One of the common difficulties in scientific research is the determination of sample size. Calculations of the sample size are necessary to validate the test designs and justify the authenticity of the test results. The determination of the sample size for testing is a key element. There are many situations where sample size is determined depending on the specificity of the study and the potential difficulties in recruiting participants. In some situations involving epidemiological studies, difficulties in recruiting the study group arise from the need to recruit in temporary patient locations. In the case of the study presented here, these were hemodialysis situations. Difficulties resulting from such a condition were related to the limited comfort of performing the dental examination, the necessity to prepare portable diagnostic kits and sterile packs in advance, and the inability to perform radiological diagnostic imaging. Another difficulty may be the necessity to perform examinations at times of the day/night sanctioned by the dialysis cycle.

### 4.8. Future Research Plan

The results of the present study should be considered important for the assessment of the condition and for improvement in the quality of life of multimorbid adult patients diagnosed with end-stage chronic kidney disease. In the further planned stages of the study, it will be important to correlate the results of the dental examination and the observations made by the general specialist medical teams in order to provide the necessary long-term care for this group of patients in the future with interdisciplinary teams.

## 5. Conclusions

A positive correlation of chronic diseases with the frequency of occurrence of oral pathologies can be observed, especially those that are dependent on saliva deficiency. Undoubtedly, this influences the quality of life of multimorbid patients who have to struggle with irreversible changes in their lives. All these factors make it necessary to provide this group of people long-term care with interdisciplinary teams.

## Figures and Tables

**Table 1:** Frequency and percentage distribution of answers to the questions regarding xerostomia.

Question	Answer	Number of Answers (Percentage Distribution)					*p*
		CG	R	R + H	R + D	R + H + D	
I drink water while swallowing the food.	never	22 (46%)	13 (31%)	24 (30%)	5 (31%)	11 (26%)	0.007
	sometimes	20 (42%)	18 (43%)	25 (32%)	7 (44%)	9 (21%)	
	often	6 (13%)	11 (26%)	30 (38%)	4 (25%)	23 (53%)	
I feel dryness in oral cavity while eating meals.	never	26 (54%)	24 (57%)	36 (46%)	6 (38%)	25 (58%)	0.101
	sometimes	17 (35%)	11 (26%)	17 (22%)	6 (38%)	8 (19%)	
	often	5 (10%)	7 (17%)	26 (33%)	4 (25%)	10 (23%)	
Feeling of dryness wakes me up and I drink at night.	never	23 (48%)	20 (48%)	23 (29%)	9 (56%)	15 (35%)	0.176
	sometimes	17 (35%)	14 (33%)	28 (35%)	3 (19%)	15 (35%)	
	often	8 (17%)	8 (19%)	28 (35%)	4 (25%)	13 (30%)	
I feel discomfort while eating dry meals.	never	27 (56%)	24 (57%)	41 (52%)	10 (63%)	19 (44%)	0.242
	sometimes	12 (25%)	12 (29%)	15 (19%)	1 (6%)	8 (19%)	
	often	9 (19%)	6 (14%)	23 (29%)	5 (31%)	16 (37%)	
I suck candies to decrease the feeling of dryness.	never	39 (81%)	24 (57%)	34 (43%)	6 (38%)	26 (60%)	0.001
	sometimes	6 (13%)	11 (26%)	17 (22%)	7 (44%)	9 (21%)	
	often	3 (6%)	7 (17%)	28 (35%)	3 (19%)	8 (19%)	
I have difficulties in swallowing food.	never	40 (83%)	31 (74%)	56 (71%)	11 (69%)	32 (74%)	0.120
	sometimes	8 (17%)	9 (21%)	10 (13%)	2 (13%)	6 (14%)	
	often	0 (0%)	2 (5%)	13 (16%)	3 (19%)	5 (12%)	
My skin on the face is dry.	never	21 (44%)	20 (48%)	23 (29%)	5 (31%)	18 (42%)	0.268
	sometimes	19 (40%)	12 (29%)	26 (33%)	5 (31%)	13 (30%)	
	often	8 (17%)	10 (24%)	30 (38%)	6 (38%)	12 (28%)	
My eyes are dry.	never	34 (71%)	27 (64%)	39 (49%)	5 (31%)	25 (58%)	0.006
	sometimes	8 (17%)	11 (26%)	14 (18%)	8 (50%)	9 (21%)	
	often	6 (13%)	4 (10%)	26 (33%)	3 (19%)	9 (21%)	
My lips are dry.	never	27 (56%)	24 (57%)	29 (37%)	6 (38%)	20 (47%)	0.084
	sometimes	14 (29%)	14 (33%)	24 (30%)	6 (38%)	10 (23%)	
	often	7 (15%)	4 (10%)	26 (33%)	4 (25%)	13 (30%)	

**Table 2:** Frequency and percentage distribution of answers to the questions regarding feeling of thirst.

Question	Answer	Number of Answers (Percentage Distribution)					*p*
		CG	R	R + H	R + D	R + H + D	
I often feel thirst, what is problematic.	never	32 (67%)	13 (31%)	27 (34%)	8 (50%)	14 (33%)	0.000
	sometimes	14 (29%)	17 (40%)	17 (22%)	5 (31%)	11 (26%)	
	often	2 (4%)	12 (29%)	35 (44%)	3 (19%)	18 (42%)	
I feel thirst during the day.	never	16 (33%)	10 (24%)	11 (14%)	4 (25%)	6 (14%)	0.014
	sometimes	23 (48%)	21 (50%)	28 (35%)	6 (38%)	17 (40%)	
	often	9 (19%)	11 (26%)	40 (51%)	6 (38%)	20 (47%)	
I feel thirst during the night.	never	31 (65%)	21 (50%)	29 (37%)	7 (44%)	18 (42%)	0.031
	sometimes	12 (25%)	15 (36%)	25 (32%)	6 (38%)	10 (23%)	
	often	5 (10%)	6 (14%)	25 (32%)	3 (19%)	15 (35%)	
I take thirst into consideration when planning my free time.	never	43 (90%)	29 (69%)	39 (49%)	9 (56%)	26 (60%)	0.000
	sometimes	4 (8%)	11 (26%)	20 (25%)	5 (31%)	7 (16%)	
	often	1 (2%)	2 (5%)	20 (25%)	2 (13%)	10 (23%)	
I feel thirst before hemodialysis.	never	-	25 (60%)	39 (49%)	7 (44%)	24 (56%)	0.431
	sometimes	-	10 (24%)	13 (16%)	4 (25%)	10 (23%)	
	often	-	7 (17%)	27 (34%)	5 (31%)	9 (21%)	
I feel thirst during hemodialysis.	never	-	26 (62%)	41 (52%)	8 (50%)	20 (47%)	0.762
	sometimes	-	9 (21%)	22 (28%)	3 (19%)	13 (30%)	
	often	-	7 (17%)	16 (20%)	5 (31%)	10 (23%)	
I feel thirst after hemodialysis.	never	-	20 (48%)	24 (30%)	7 (44%)	17 (40%)	0.451
	sometimes	-	10 (24%)	21 (27%)	4 (25%)	7 (16%)	
	often	-	12 (29%)	34 (43%)	5 (31%)	19 (44%)	

**Table 3:** *Kruskal–Wallis* test results regarding saliva pH.

	*Kruskal–Wallis* Test Results		
	X^2^	*p*	ε^2^
[H^+^]	0.34	0.987	0.00

**Table 4:** The results of *chi-squared* test for pathologies observed in mucosa.

Pathology	Chi-Squared Test Results			
	X^2^	df	*p*	η
Pain	2.39	4	0.664	0.10
Burning mouth syndrome	3.44	4	0.487	0.12
Dry mouth syndrome	17.06	4	0.002	0.27
Taste disorders	4.60	4	0.331	0.14
Ulcerations	4.35	4	0.360	0.14
White patches	4.25	4	0.373	0.14
Red patches	1.47	4	0.831	0.08
Malformations	3.47	4	0.482	0.12
Ecchymosis	14.72	4	0.005	0.25
*Candidiasis*	15.77	4	0.003	0.26
Herpes simplex	7.49	4	0.112	0.18
Geographic tongue	1.56	4	0.816	0.08
Fissured tongue	24.62	4	0.000	0.33
Halitosis	6.00	4	0.199	0.16
Signs of previous operations	1.89	4	0.755	0.09
Trauma-related lesions	11.56	4	0.021	0.23
Overgrowth gingiva	2.18	4	0.703	0.10

## Data Availability

The data presented in this study are available on request from the corresponding author.
